# Combining estimates of interest in prognostic modelling studies after multiple imputation: current practice and guidelines

**DOI:** 10.1186/1471-2288-9-57

**Published:** 2009-07-28

**Authors:** Andrea Marshall, Douglas G Altman, Roger L Holder, Patrick Royston

**Affiliations:** 1 Centre for Statistics in Medicine, University of Oxford, Oxford, UK; 2 Warwick Clinical Trials Unit, University of Warwick, Coventry, UK; 3 Department of Primary Care & General Practice, University of Birmingham, Birmingham, UK; 4 Hub for Trials Methodology Research and UCL, MRC Clinical Trials Unit, London, UK

## Abstract

**Background:**

Multiple imputation (MI) provides an effective approach to handle missing covariate data within prognostic modelling studies, as it can properly account for the missing data uncertainty. The multiply imputed datasets are each analysed using standard prognostic modelling techniques to obtain the estimates of interest. The estimates from each imputed dataset are then combined into one overall estimate and variance, incorporating both the within and between imputation variability. Rubin's rules for combining these multiply imputed estimates are based on asymptotic theory. The resulting combined estimates may be more accurate if the posterior distribution of the population parameter of interest is better approximated by the normal distribution. However, the normality assumption may not be appropriate for all the parameters of interest when analysing prognostic modelling studies, such as predicted survival probabilities and model performance measures.

**Methods:**

Guidelines for combining the estimates of interest when analysing prognostic modelling studies are provided. A literature review is performed to identify current practice for combining such estimates in prognostic modelling studies.

**Results:**

Methods for combining all reported estimates after MI were not well reported in the current literature. Rubin's rules without applying any transformations were the standard approach used, when any method was stated.

**Conclusion:**

The proposed simple guidelines for combining estimates after MI may lead to a wider and more appropriate use of MI in future prognostic modelling studies.

## Background

Prognostic models play an important role in the clinical decision making process as they help clinicians to determine the most appropriate management of patients. A good prognostic model can provide an insight into the relationship between the outcome of patients and known patient and disease characteristics [1,2].

Missing covariate data and censored outcomes are unfortunately common occurrences in prognostic modelling studies [3], which can complicate the modelling process. Multiple imputation (MI) is one approach to handle the missing covariate data that can properly account for the missing data uncertainty [4]. Missing values are replaced with *m *(>1) values to give *m *imputed datasets. Previously, three to five imputations were considered sufficient to give reasonable efficiency provided that the fraction of missing information is not excessive [4]. However, with increased computer capabilities, the limitations on *m *have diminished and therefore it may be more sensible to use 20 [5] or more [6] imputations. The imputation model, used to generate plausible values for the missing data, should contain all variables to be subsequently analysed including the outcome and any variables that help to explain the missing data [7]. Outcome tends to be incorporated into the imputation model by including both the event status, indicating whether the event, i.e. death, has occurred or not, and the survival time, with the most appropriate transformation [7]. Due to censoring, this approach is not exact and may introduce some bias, but should still help to preserve important relationships in the data. The *m *imputed datasets are each analysed using standard statistical methods. The estimates from each imputed dataset must then be combined into one overall estimate together with an associated variance that incorporates both the within and between imputation variability [4]. Rubin [4] developed a set of rules for combining the individual estimates and standard errors (SE) from each of the *m *imputed datasets into an overall MI estimate and SE to provide valid statistical results, which will be described in the methods section. These rules are based on asymptotic theory [4]. It is assumed that complete data inferences about the population parameter of interest (*Q*) are based on the normal approximation , where  is a complete data estimate of *Q *and *U *is the associated variance for [4]. In a frequentist analysis,  would be a maximum likelihood estimate of *Q*, *U *the inverse of the observed information matrix and the sampling distribution of  is considered approximately normal with mean *Q *and variance *U *[8]. From a Bayesian perspective,  and associated variance *U *should approximate to the posterior mean and variance of *Q *respectively, under a reasonable complete data model and prior [9]. Inference is based on the large sample approximation of the posterior distribution of *Q *to the normal distribution [8]. With missing data, estimates of the parameters of interest are calculated on each of the *m *imputed datasets to give  with associated variances *U*_1_,..., *U*_*m*_. Provided that the imputation procedure is proper [4], thus reflecting sufficient variability due to the missing data, and samples are large, the overall MI estimate and variance approximate the mean and variance of the posterior distribution of *Q *[6,8]. The overall MI estimators and confidence intervals would be improved if combined on a scale where the posterior of *Q *is better approximated by the normal distribution [6,10,11]. When the normality assumption appears inappropriate for estimates of the parameters of interest, suitable transformations that make the normality assumption more applicable should be considered [4]. In circumstances where transformations cannot be identified, alternative robust summary measures [12], such as medians and ranges, may provide better results than applying Rubin's rules. In the context of prognostic modelling, there are no explicit guidelines for handling estimates of the parameters of interest after MI, such as predicted survival probabilities and assessments of model performance, where it is unclear whether simply applying Rubin's rules is appropriate.

Example techniques and parameters of interest in prognostic modelling studies and the rules currently available for combining estimates after MI are summarised. This paper will then provide guidelines on how estimates of the parameters of interest in prognostic modelling studies can be combined after performing MI. A review of the current practice for combining estimates after MI within published prognostic modelling studies is provided.

## Methods

### Prognostic models

Prognostic models, focusing on time to event data that may be censored, are often constructed using survival analysis techniques such as the Cox proportional hazards model or parametric survival models. Ideally, pre-specification of the covariates prior to the modelling process, and hence fitting the full model results in more reliable and less biased prognostic models than data derived models based on statistical significance testing [13]. Such a model can be as large and complex as permitted by the number of observed events [13,14].

The parameters of interest in prognostic modelling are summarised in Table 1. These usually include the regression coefficients or the hazard ratio for each covariate in the model and their associated significance in the model. Assessments of the model performance, for example model fit, predictive accuracy, discrimination and calibration are also important issues in prognostic modelling studies.

**Table 1:** Parameter of interest in prognostic modelling studies and ways to combine estimates after MI

Parameters	Possible methods for combining estimates of parameters after MI*
**Covariate distribution**	
Mean Value	Rubin's rules
Standard Deviation	Rubin's rules
Correlation	Rubin's rules after Fisher's Z transformation

**Model parameters**	
Regression coefficient	Rubin's rules
Hazard ratio	Rubin's rules after logarithmic transformation
Prognostic Index/linear predictor per patient	Rubin's rules

**Model fit and performance**	
Testing significance of individual covariate in model	Rubin's rules using a Wald test for a single estimates (Table 2(A))
Testing significance of all fitted covariates in model	Rubin's rules using a Wald test for multivariate estimates (Table 2(B))
Likelihood ratio *χ*^2 ^test statistic	Rules for combining likelihood ratio statistics if parametric model (Table 2(D)) or *χ*^2 ^statistics if Cox model (Table 2(C))
Proportion of variance explained (e.g. R^2 ^statistics)	Robust methods
Discrimination (c-index)	Robust methods
Prognostic Separation D statistic	Rubin's rules
Calibration (Shrinkage estimate)	Robust methods

**Prediction**	
Survival probabilities	Rubin's rules after complementary log-log transformation
Percentiles of a survival distribution	Rubin's rules after logarithmic transformation

* Reflect the authors' experiences and current evidence.

The likelihood ratio chi-square (*χ*^2^) statistic tests the hypothesis of no difference between the null model given a specified distribution and the fitted prognostic model with *p *parameters [15]. Various proportion of explained variance measures have been proposed as measures of the goodness of fit and predictive accuracy (e.g. by Schemper and Stare [16], Schemper and Hendersen [17], O'Quigley, Xu and Stare [18] and Nagelkerke's R^2 ^[15]). However, no approach is completely satisfactory when applied to censored survival data. Discrimination assesses the ability to distinguish between patients with different prognoses, which can be assessed using the concordance index (c-index) [19] or alternatively using the prognostic separation D statistic [20]. Calibration determines the extent of the bias in the predicted probabilities compared to the observed values. A shrinkage estimator provides a measure of the amount needed to recalibrate the model to correctly predict the outcome of future patients using the fitted model [21]. The prognostic model is often summarised by reporting the predictive survival probabilities at specific time-points of interest or quantiles of the survival distribution for each prognostic risk group.

### Rules for MI inference

The rules developed by Rubin [4] for combining either a single estimate or multiple estimates from each imputed dataset into an overall MI estimate and associated SE will be summarised. Performing hypothesis testing for a single estimate or based on multiple estimates will be described together with the extensions for combining *χ*^2 ^statistics [8] and likelihood ratio *χ*^2 ^statistics [22].

### Combining parameter estimates

For a single population parameter of interest, *Q*, e.g. a regression coefficient, the MI overall point estimate is the average of the *m *estimates of *Q *from the imputed datasets, [4]. The associated total variance for this overall MI estimate is , where  is the estimated within imputation variance and  is the between imputation variance [4]. Inflating the between imputation variance by a factor 1/*m *reflects the extra variability as a consequence of imputing the missing data using a finite number of imputations instead of an infinite number of imputations [4]. When *B *dominates  greater efficiency, and hence more accurate estimates, can be obtained by increasing *m*. Conversely, when  dominates *B*, little is gained from increasing *m *[4].

These procedures for combining a single quantity of interest can be extended in matrix form to combine *k *estimates of parameters, e.g. *k *regression coefficients, where  is a *k *× 1 vector of these estimates and **U **is the associated *k *× *k *covariance matrix [4].

### Hypothesis testing

#### Significance level based on a single combined estimate

A significance level for testing the null hypothesis that a single combined estimate equals a specific value, *Q*_0_: H_0_: *Q *= *Q*_0 _can be obtained using a Wald test by comparing the test statistic  against the F distribution with one and *v *= (*m *- 1)(1 + *r*^-1^)^2 ^degrees of freedom, where  is the relative increase in variance due to the missing data (Table 2(A)) [4]. When the degrees of freedom, *v*, are close to the number of imputations performed, for example with a large fraction of missing information about the parameter of interest, then estimates of the parameter may be unstable and more imputations are required.

**Table 2:** Summary of significance tests for combining different estimates from *m *imputed datasets after MI

Estimate	*F*	Test statistic	Degrees of freedom (df)	Relative increase in variance (*r*)
**A) Scalar **	*F*_1, *v*_	, H_0_: *Q *= *Q*_0_	*v *= (*m *- 1)(1 + *r*^-1^)^2^	

**B) Multivariate **		H_0_:**Q = Q**_0_, *k *= number of parameters	where *a *= *k*(*m *- 1)	

**C)** *χ*^2 ^**statistics** *w*_1_,..., *w*_*m*_		*k *= df associated with *χ*^2 ^tests		

**D) Likelihood Ratio ***χ*^2 ^**statistics** *w*_*L*1_,..., *w*_*Lm*_		*k *= number of parameters in fitted model	where *a *= *k*(*m *- 1)	

**KEY**: *F *= value from the F-distribution, which the test statistic is compared to.

= average of the *m *imputed data estimates.

= within imputation variance.

*B *= between imputation variance.

*T *= total variance for the combined MI estimate.

*w*_*j*_, *j *= 1,..., *m *= *χ*^2 ^statistics associated with testing the null hypothesis *H*_*o *_: *Q *= *Q*_*o *_on each imputed dataset, such that the significance level for the *j*^*th *^imputed dataset is *P*{ > *w*_*j*_}, where  is the *χ*^2 ^value with *k *degrees of freedom (Rubin 1987).

= average of the repeated *χ*^2 ^statistics.

= average of the *m *likelihood ratio statistics, *w*_*L*1_,..., *w*_*Lm*_, evaluated using the average MI parameter estimates and the average of the estimates from a model fitted subject to the null hypothesis.

#### Significance level based on combined multivariate estimates

In the context of prognostic modelling, it is useful to test the global null hypothesis that all *k *regression estimates are a specific value, zero say. A significance level for testing the hypothesis that the combined MI estimate, , equals a particular vector of values **Q**_*o *_is provided in Table 2(B)[9]. This ideal approach using a Wald test requires a vector of point estimates and a covariance matrix to be stored from each imputed dataset, which can be cumbersome for large *k*, as can result from fitting categorical variables in the regression model.

#### Significance level based on combining χ^2 ^statistics

An alternative to testing the multivariate point estimates is the method for combining *χ*^2 ^statistics, associated with testing a null hypothesis of *H*_*o *_: *Q *= *Q*_*o*_, e.g. a regression coefficient is zero or all regression coefficients are zero (Table 2(C)) [8]. This approach is useful when there are a large number of parameters to estimate, the full covariance matrix is unobtainable from standard software or too large to store, or only the *χ*^2 ^statistics are available. This approach is deficient compared to the method for combining multivariate estimates and should be used only as a guide, especially when there are a large number of parameters compared to only a small number of imputations [22]. The true p-value lies between a half and twice this calculated value [8]. A considerable amount of information is wasted from only using the *χ*^2 ^statistics and thus there is a consequent loss of power [22]. This approach may be improved by multiplying the relative increase in variance estimate (*r*_2 _in Table 2(C)) by a factor  representing the number of model parameters. Justification for this adjustment lies in the fact that each *χ*^2 ^statistic is based on *k *degrees of freedom, but unlike the other approaches, this is not accounted for in the relative increase in variance calculations originally proposed by Li et al. [8].

#### Significance level based on combining likelihood ratio χ^2 ^statistics

The method for combining the likelihood ratio *χ*^2 ^statistics [22] from each imputed dataset is used to obtain an overall significance level for testing the hypothesis of no difference between two nested prognostic models (Table 2(D)). This is an intermediate approach between combining multivariate estimates and combining *χ*^2 ^statistics. The obtained significance level should be asymptotically equivalent to that based on the combined multivariate estimates [22].

The likelihood function needs to be fully specified in order to calculate the likelihood ratio statistics determined at the average of the parameter estimates over the *m *imputations from fitting the regression model either subject to the null hypothesis or the alternative hypothesis with covariates included. This may be difficult for the Cox proportional hazards model, which uses the partial likelihood function.

### Guidelines for combining estimates of interest in prognostic studies

The procedures for combining multiply imputed estimates that are of particular interest in prognostic modelling are discussed in the following subsections. It is assumed that the full prognostic model is fitted and its performance evaluated within each imputed dataset and the required estimates (as given in Table 1) obtained. The estimates of the parameters of interest (Table 1) are separated into those where the Rubin's rules for MI inference can be applied, those where suitable transformations can be found to improve normality and those where suitable transformations cannot be identified and therefore alternative summary measures are proposed.

#### Combining estimates using Rubin's rules

The sample mean of a covariate, standard deviation, regression coefficients, individual prognostic index and the prognostic separation estimates can all be combined using Rubin's rules for single estimates. It is important to emphasise that the variance associated with a sample mean of a covariate is the sample variance divided by the number of observations and hence not just its sample variance [9]. The standard deviation of the data can be treated like any other parameter to give a more appropriate and efficient combined MI estimate than reporting the standard deviation from only one imputed dataset. The regression coefficients, and hence the prognostic separation D statistic, from fitting either a Cox proportional hazards or a Weibull model should be asymptotically normal at least with large samples [15], thus making Rubin's rules appropriate.

The likelihood ratio statistic for testing the hypothesis of no difference between two nested prognostic models from each imputed dataset can be combined using the inferences for likelihood ratio statistics (Table 2(D)), provided that the log-likelihood function can be fully specified, e.g. for fully parametric models such as the Weibull model. The Cox proportional hazards model uses the partial likelihood function as the baseline hazards are unspecified, and therefore can be more difficult to specify. Hence, it may be easier to use the less precise approach for combining *χ*^2 ^statistics (Table 2(C)). However, both these approaches are less accurate than testing the significance of the model using a Wald test based on the combined multivariate regression parameter estimates (Table 2(B)). The latter approach may be considered the preferred approach, when possible.

#### Combining estimates using Rubin's rules after suitable transformation

The correlation coefficient, hazard ratios, predicted survival probabilities and percentiles of the survival distribution can all be combined using Rubin's rules after suitable transformations to improve normality. The obtained combined estimates should be back transformed onto their original scale prior to analysis.

Fisher's z transformation [23] provides a suitable transformation for the sample correlation coefficient, which has an approximate normal distribution [9]. For large samples, the log hazard ratio from a survival model, which is simply the regression coefficient, is approximately normally distributed and therefore should be used. A more extreme pooled estimate than appropriate would be obtained if the hazard ratio was not transformed, as the estimates would be averaged over the posterior medians and not the posterior means as required.

The complementary log-log transformation for the predicted survival probability at particular time-points gives a possible range of (-∞, +∞) instead of the survivorship estimate being bounded by zero and one, and is often used to determine reasonable confidence intervals [24]. A suitable transformation for the survival time associated with the *p*^*th *^percentile of a survival distribution is the logarithmic transformation, as this gives a possible range of (-∞, +∞) instead of being bounded by zero and infinity and is generally used to obtain a confidence interval [25]. Estimates for the predicted survival probabilities at specific time-points, e.g. at 2 years, or survival times at particular percentiles can be obtained within each imputed dataset for the average covariate values, provided that researchers acknowledge that this does not represent the diversity of the patients in the sample [26]. Alternatively, predicted survival probabilities can be obtained for specific covariate patterns or for an individual patient.

#### Combining model performance measures where the normality assumption is uncertain and variance estimates are generally unavailable

When considering model performance measures, the imputation model should be more general than the prognostic models being investigated, as the performance measures are more sensitive to the choice of imputation model and therefore may produce more bias than seen in the regression parameter estimates from the prognostic model. If one is willing to accept the large sample approximation to normality for the proportion of variance explained measures, e.g. Nagelkerke's R^2 ^statistic [15], the c-index and the shrinkage estimator, then these estimates can be simply treated as another estimate that can be averaged using Rubin's rules for single estimates. However, estimates for these measures are generally bounded by zero and one, not symmetrically distributed and do not necessarily follow a specific distribution, so are unlikely to follow a normal distribution. Therefore the standard MI techniques for combining into one estimate, even after applying a transformation, may not provide the best estimate. In addition, an overall MI variance incorporating sufficient uncertainty cannot be determined as variance estimates associated with these performance measures are generally unavailable. The lack of a within imputation variance estimate also restricts the use of sophisticated robust location and scale estimators, such as the M-estimators [12]. The median, inter-quartile range or full range of the *m *estimates may provide a more appropriate reflection of the distribution of the values over the imputed datasets, as reported by Clark and Altman [27] and Sinharay, Stern and Russell [28] for the R^2 ^statistics and by Clark and Altman [27] for the c-index. Using the median absolute deviation [12] could provide an alternative measure of the dispersion of values around the median.

## Methods for literature review

A literature search was performed within the PubMed (National Library of Medicine) and Web of Science^® ^bibliographic software of all articles published before June 2008 that used multiple imputation techniques and a survival analysis to obtain a prognostic model. Methodological papers were excluded. The aim of the review was to identify how estimates of the parameters of interest in prognostic modelling studies have been combined after performing MI in the published literature.

## Results

Sixteen non-methodological articles were identified. The MI techniques reported were varied with no overall consensus on technique or statistical software. The number of imputations ranged from five to 10000, with the majority of studies using five or ten imputations. The amount of missingness reported also varied from studies with relatively little missing data [29] to those with large amounts of missingness [30].

In seven articles, no mention of how the estimates of interest were combined after MI was given. Clark et al. [30] reported pooled summary estimates from the imputed datasets and Rouxel et al. [31] stated that "the multivariable analysis took into account the potential multiple imputation". Although neither article provided any details or references, Rubin's rules were presumably used. The remaining seven studies reported that Rubin's rules [4] were used to combine the estimates of interest after fitting a variety of regression models, such as a Cox regression model [29,32-34], multiple Poisson regression models [35] or a Weibull model [36,37]. The estimates reported in the published literature were predominately the regression coefficients and associated SEs, hazard ratios and 95% confidence limits, and significance of the individual covariates in the model. The estimates also included combining percentiles from the Weibull survival distribution [36] and the median survival time and associated 95% confidence intervals from the Cox model [32] using Rubin's rules. No details of any transformations applied to these estimates prior to using Rubin's rules were reported. Gill et al. [29] and Clark et al. [30] reported model performance measures after MI, but did not explicitly state how this was achieved after MI.

## Discussion

With the advances in computer technologies and software, MI is becoming more accessible. MI has been performed prior to the analysis of several prognostic modelling studies, e.g. [30,31]. Few published studies explicitly stated how the reported results were obtained after MI. None of the articles identified within the current review reported that transformations were applied prior to applying Rubin's rules for any of the estimates.

This paper has suggested guidelines for combining multiply imputed estimates that are of interest when a survival model is fitted to a dataset and suitable performance measures and predicted survival probabilities are required for summarising the model (Table 1). These proposed guidelines are based on our own experiences and current evidence, although evidence for the appropriateness for some parameters of interest such as the mean and regression coefficients are more widely available than for others such as the model performance measures. Following these guidelines can provide a more uniform approach for handling these estimates in future studies and hence comparability of reported estimates between similar studies. The standard Rubin's rules [4] should be applied to the estimates where the asymptotic normality assumption holds or where suitable transformations can be found. When the asymptotic normality assumption does not appear to hold or is not easily achievable, the average estimate and associated variance may be unsuitable especially with highly skewed distributions, as this could give undue weight to the tails of the distribution. Median and ranges may be more suitable, e.g. for some model performance measures, where variance estimates are generally unavailable. More sophisticated robust estimators, such as the robust M-estimators [12], may be useful when a within imputation variance can be easily calculated. However, these robust techniques are not likelihood based, as is the case with Rubin's rules. Harel [38] showed that the proportion of variation explained measures, R^2^, from a linear regression model fitted to normally distributed data can be considered as a squared correlation coefficient and can be transformed by taking the square root and then applying Fisher's Z transformation as for the correlation coefficient. However whether this approach would apply to R^2 ^measures from a survival regression model that may be affected by censored observations as arises in survival analysis is debatable and therefore robust methods are recommended here.

In this paper, model performance measures were calculated within each imputed dataset using the constructed prognostic model for that dataset and then combined to give an overall multiply imputed measure. The performance of a prognostic model derived using a development sample will also need to be externally validated using an independent dataset [1], but missing data within the development and or validation sample complicate these analyses. At present there are no clear guidelines on the appropriate handling of missing data and the use of MI when externally validating a prognostic model and therefore further research is required through the use of simulation studies. The extension to constructing prognostic models using variable selection procedures with multiply imputed datasets provides an added complexity, which also requires further investigation. One possible solution is to perform backwards elimination by fitting the full model in each imputed dataset and using the combined estimates to determine the least significant variable for which to exclude and then refit this reduced model. This process is continued until all non-prognostic variables have been eliminated [27]. Alternatively, bootstrapping could be incorporated [39] or a model averaging approach, as considered within the Bayesian framework [40], may also be possible.

## Conclusion

The review of current practice highlighted deficiencies in the reporting of how the multiply imputed estimates given in the published articles were obtained. Thus, it is recommended that future studies include a more thorough description of the methods used to combine all estimates after MI.

The ability to use MI methods that are readily available in standard statistical software and apply simple rules to combine the estimates of interest rather than requiring problem specific programmes makes MI more accessible to practising statistician. We hope that this may lead to a more widespread and appropriate use of MI in future prognostic modelling studies and improved comparability of the obtained estimates between studies.

## Abbreviations

MI: multiple imputation; m: number of imputations; SE: standard error.

## Competing interests

The authors declare that they have no competing interests.

## Authors' contributions

All authors made substantial contributions to the ideas presented in this manuscript. AM participated in the conception of this research, the methodological content, the design, coordination and analysis of the literature review and drafted the manuscript. DGA was involved in the conception and design of the study and helped in the writing of the manuscript. RLH participated in the design and methodological content of this study and in the revision of the manuscript. PR contributed to the methodological content of this research and the revision of the manuscript. All authors have read and approved the final manuscript.

## Pre-publication history

The pre-publication history for this paper can be accessed here:

http://www.biomedcentral.com/1471-2288/9/57/prepub
